# Supplementary material for: Open-Access 12-Minute MRI Screening for Acute Appendicitis: A Five-Year Retrospective Observational Study of Diagnostic Accuracy
Source: J Clin Med. 2024 Nov 28;13(23):7257. doi: 10.3390/jcm13237257 (PMC11642492; doi:10.3390/jcm13237257)
Supplement: Supplementary file 1 [file jcm-13-07257-s001.zip › jcm-3336944-supplementary.pdf]

Supplementary Table S1. Prevalence Threshold Formula by TP, FP, TN, FN.

Published Formula [10]:

$$\textbf{Prevalence Threshold} = \frac{\sqrt{a(-b+1)} + b - 1}{\varepsilon - 1}$$

a = Sensitivity; b = Specificity; ε = Specificity + Sensitivity

Input:

$$\text{Sensitivity} = \left(\frac{TP}{TP + FN}\right)$$

$$\text{Specificity} = \left(\frac{TN}{FP + TN}\right)$$

$$\left(\frac{FP}{FP + TN}\right) + \left(\frac{TN}{FP + TN}\right) = 1$$

$$\left(\frac{TN}{FP + TN}\right) - 1 = -\left(\frac{FP}{FP + TN}\right)$$

$$\left(\frac{FP}{FP + TN}\right) = 1 - \left(\frac{TN}{FP + TN}\right)$$

Substitutions:

$$\frac{\sqrt{a(-b+1)} + b - 1}{\varepsilon - 1} = \frac{\sqrt{\left(\frac{TP}{TP + FN}\right)\left(-\left(\frac{TN}{FP + TN}\right) + 1\right)} + \left(\frac{TN}{FP + TN}\right) - 1}{\left(\frac{TP}{TP + FN}\right) + \left(\frac{TN}{FP + TN}\right) - 1}$$

$$\frac{\sqrt{a(-b+1)} + b - 1}{\varepsilon - 1} = \frac{\sqrt{\left(\frac{TP}{TP + FN}\right)\left(1 - \left(\frac{TN}{FP + TN}\right)\right)} + -\left(\frac{FP}{FP + TN}\right)}{\left(\frac{TP}{TP + FN}\right) + -\left(\frac{FP}{FP + TN}\right)}$$

$$\frac{\sqrt{a(-b+1)} + b - 1}{\varepsilon - 1} = \frac{\sqrt{\left(\frac{TP}{TP + FN}\right)\left(\frac{FP}{FP + TN}\right)} + -\left(\frac{FP}{FP + TN}\right)}{\left(\frac{TP}{TP + FN}\right) + -\left(\frac{FP}{FP + TN}\right)}$$

Ergo:

$$\textbf{Prevalence Threshold} = \frac{\sqrt{\left(\frac{TP}{TP + FN}\right)\left(\frac{FP}{FP + TN}\right)} - \left(\frac{FP}{FP + TN}\right)}{\left(\frac{TP}{TP + FN}\right) - \left(\frac{FP}{FP + TN}\right)}$$
